# Towards subsidized malaria rapid diagnostic tests. Lessons learned from programmes to subsidise artemisinin-based combination therapies in the private sector: a review

**DOI:** 10.1093/heapol/czv028

**Published:** 2015-04-09

**Authors:** Cristina Lussiana

**Affiliations:** Population Services International, Morro Bento, Luanda, Angola.

**Keywords:** Affordable Medicines Facility-malaria (AMFm), artemisinin-based combination therapies (ACTs), subsidy, malaria, rapid diagnostic tests (RDTs)

## Abstract

The idea of a private sector subsidy programme of artemisinin-based combination therapies (ACTs) was first proposed in 2004. Since then, several countries around the world have hosted pilot projects or programmes on subsidized ACTs and/or the Affordable Medicines Facility-malaria programme (AMFm). Overall the private sector subsidy programmes of ACTs have been effective in increasing availability of ACTs in the private sector and driving down average prices but struggled to crowd out antimalarial monotherapies. The results obtained from this ambitious strategy should inform policy makers in the designing of future interventions aimed to control malaria morbidity and mortality. Among the interventions recently proposed, a subsidy of rapid diagnostic tests (RDTs) in the private sector has been recommended by governments and international donors to cope with over-treatment with ACTs and to delay the emergence of resistance to artemisinin. In order to improve the cost-effectiveness of co-paid RDTs, we should build on the lessons we learned from almost 10 years of private sector subsidy programmes of ACTs in malaria-endemic countries.

**Key Messages**

- Overall the private sector subsidy programmes were effective in increasing availability of ACTs in the private sector and lowering average prices of ACTs in many malaria endemic countries. They have struggled to crowd out antimalarial monotherapies and to improve access to ACTs in remote areas. The private sector subsidy programmes of ACTs greatly increased the number of ACTs courses procured by the private sector of several countries.
- Only a low percentage of the subsidized ACTs were given upon confirmatory diagnosis of malaria. In order to link malaria treatment with a positive diagnosis for malaria, a subsidy of rapid diagnostic tests (RDTs) in the private sector has been advocated and recommended by governments and international donors. This policy will reduce unnecessary sales of ACTs and decrease the chances of developing resistance to artemisinin from *Plasmodium falciparum*.
- In order to improve the cost-effectiveness of a subsidy of RDTs, we should build on the lessons we learned from almost 10 years of private sector subsidy programmes of ACTs in the private sector of malaria-endemic countries.

## Introduction

In the past decade, the documented growing resistance of *Plasmodium falciparum* to chloroquine has led to the introduction of other effective antimalarial treatments for chloroquine-resistant cases [World Health Organization (WHO) 2001, 2006]. Among those, artemisinin-based combination therapies (ACTs) have gained in popularity. They consist of artemisinin derivatives (artemisinins) from the plant *Artemisia annua*, including dihydroartemisinin, artesunate and artemether, which are combined with other effective drugs such as lumefantrine, amodiaquine, mefloquine or sulfadoxine-pyrimethamine (SP). ACTs rapidly became the only effective antimalarial treatments to treat uncomplicated malaria in regions, where *P. falciparum* showed high resistance to chloroquine and artemether-lumefantrine (AL) was the first ACT to be recommended by the World Health Organization (WHO) for treatment of uncomplicated malaria caused by *P. falciparum* (WHO 2001).

In 2001, the WHO released a policy aimed at increasing the use of ACTs all over the world and delaying the emergence of resistance to artemisinin by *P. falciparum* (WHO 2001). Starting in 2002, several ministries of health (MoH) in malaria-endemic countries adopted ACTs as first-line antimalarial treatments for uncomplicated malaria and made them available in public health facilities (HFs; WHO 2006). In 2004, the rapid increase in demand for ACTs led to a global shortage of AL from the manufacturer Novartis. Public sector demand for AL increased rapidly from 2001 to 2002, when WHO recorded orders for only 0.32 million treatment courses, to 2004–05, when the WHO and the United Nation Children Fund (UNICEF) recorded a total of 4.5 million orders. After an initial period of market instability in response to this surge, global manufacturing capacity stabilized and was able to provide the global public sector with enough ACT courses to meet the new demand. By 2007, sixty-seven countries with endemic *P. falciparum* malaria, 41 of them in Africa, had adopted ACTs as first- or second-line antimalarial treatment (Bosman and Mendis 2007).

### The need for private sector subsidy programmes of ACTs

Despite these remarkable efforts from policy-makers and MoH, providing ACTs through the public sector was not enough to increase access to ACTs to the people in need of them, as the high demand from the private sector remained unsatisfied. In 2001, the United States Agency for International Development (USAID) asked the Institute of Medicine (IOM) to provide a strategic approach for increasing availability of ACTs and at the same time to protect artemisinins for as long as possible from being lost to drug resistance (Gelband and Seiter 2007). Three years later, the IOM published a study called ‘Saving Lives, Buying Time’, which recommended a subsidy of ACTs as the most economically and biomedically efficient strategy to delay the emergence of resistance to artemisinin and to provide access to affordable and effective antimalarial treatments (Arrow *et al*. 2004). In this study, Kenneth Arrow—to date the youngest winner of the Nobel Prize in Economics, in 1972—provided the intellectual underpinning for what became, years later, the Affordable Medicines Facility-malaria programme (AMFm) (Laxminarayan *et al.* 2006; Bosman and Mendis 2007; Laxminarayan and Gelband 2009; The Global Subsidies Initiative 2010). The idea of a subsidy of ACTs serves the dual goals of reducing mortality (‘saving lives’) and delaying development of artemisinin resistance (‘buying time’) until a new active ingredient can be developed. Its main aims are:

- to reduce the cost of ACTs to between $0.20 and $0.50 per dose of adult treatment, thereby allowing millions of ACTs to be injected into the private sector,
- to increase availability and use of ACTs using funding mechanisms and competitive prices,
- to crowd out artemisinin monotherapies (AMTs) from the market by increasing the use of effective ACTs (Arrow *et al*. 2004; Laxminarayan *et al*. 2006; Laxminarayan and Gelband 2009; The Global Subsidies Initiative 2010).

**Figure 1.**
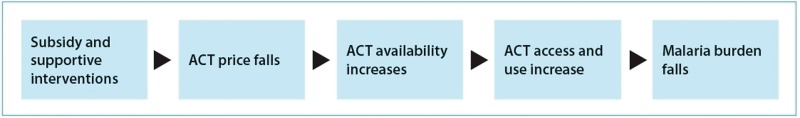
The AMFm impact model. (Schäferhoff and Yamey 2010)

The subsidy is based on a simple principle: it provides co-payments for ACTs to manufacturers, which cover most of the manufacturer’s sales price to wholesalers. Wholesalers pay a lower price for ACTs, the savings from which fall through the supply chain until the final customer. The result is an increase in affordability (Figure 1). This mechanism has lowered the price of ACTs which is now competitive with the prices of chloroquine and SP. With the incentive of a large, secure market for ACTs, wholesalers will drop their prices for the treatment course; without an assured market, potential manufacturers will not commit to adequate ACT production, nor will farmers expand the cultivation of *A. annua* (Adeyi and Atun 2009; The Global Subsidies Initiative 2010).

In order to transform the idea of a subsidy of ACTs into practice, some recommendations are called for:

- For ACTs to reach the greatest number of possible consumers, the subsidy must reduce the price of ACTs until it is closer to the price of the least expensive monotherapy available in the market;
- The subsidy has to be applied in such a way that it does not reduce access to antimalarials. In Africa, the private sector plays a key role in health-seeking behaviour, and it is estimated that up to 70% of antimalarials reach consumers through the private sector in the form of private retailers, drug outlets and informal sector;
- Because of the great variety of private-sector channels in Africa, external financing should be injected at a high point in the purchasing chain, above the level of individual countries. A subsidy near the top of the supply chain will stabilize demand and create incentives for ACT production, resulting in lower prices;
- A centralized procurement system is preferable in order to obtain competitive prices. Drugs purchased through this centralized system will be subsidized through National Malaria Control Programmes (NMCP), MoH or agencies’ partners [the President’s Malaria Initiative (PMI), international non-governmental organizations (NGOs), etc.]. Subsidized drugs injected into national private markets will reach customers at an affordable price and stimulate other producers to enter the market, thereby creating competition and ultimately pushing down the price of ACTs;
- Complementary activities are needed: regulatory bodies’ prohibition of monotherapies, behaviour change communication (BCC) campaigns to increase customers’ and providers’ knowledge of effective antimalarial treatments, malaria control interventions [such as indoor residual spraying (IRS), bed-net distributions, etc.], medical research to look for other effective antimalarial components, and a strong monitoring and evaluation (M&E) system to assess availability and pricing of antimalarial drugs (Arrow *et al*. 2004).

The strategy has led to a long-lasting and animated debate within the public health community. Bosman and Mendis (2007) stated that ‘the transition to ACTs for the treatment of malaria was probably one of the major challenges faced by malaria control in the recent past*’*. Several papers supported Bosman’s assertion, providing evidence that transforming the 2001 WHO policy into effective and widely accessible malaria treatment was hindered by two big barriers: access and price.

#### Access

It is known that most patients with fever seek treatment in the private sector (Littrell *et al.* 2011; Smith *et al*. 2011; WHO 2011a). In rural villages, most people buy malaria medication at private retailers and street sellers (Laxminarayan and Gelband 2009). In Kenya, the distance from a household to a private retailer is half the distance from a household to a public HF (Smith *et al*. 2011). The private sector’s key role in health-seeking behaviour in most Sub-Saharan populations represented a barrier to the uptake of effective ACTs in the public sector, especially in rural areas where communities bear the greatest burden of malaria (Bosman and Mendis 2007). The 2001 WHO policy did not suggest any mechanism for providing medications through the most popular channels in malaria-endemic areas, and evidence supported the concern that ACTs provided through the public sector were not reaching the right people (Laxminarayan *et al*. 2006; Medicines for Malaria Venture 2008; Kangwana *et al.* 2011). This unsatisfied need was made much worse in 2009, when the Sierra Leone Ministry of Health destroyed over 900 000 expired doses of artesunate-amodiaquine (ASAQ), valued at of over $500 000 (Amuasi *et al.* 2012).

Remarkably, years after the publication of the 2001 WHO policy, availability of AMTs and/or ineffective antimalarials was still high in the private sector (Bosman and Mendis 2007; Matowe and Adeyi 2010). In Sierra Leone, in 2009, availability of quinine was 70% in the private sector and 0% in the public sector; the situation was the opposite for the availability of ACTs (Amuasi *et al*. 2012). In Tanzania and in Uganda, in 2007, the percentage of private providers that stocked ACTs was 0 and 4%, respectively (Medicines for Malaria Venture 2008; Cohen *et al*. 2010). Also in Nigeria, public HFs were more likely than private retailers to stock AL and ASAQ (Uzochukwu *et al*. 2010). This picture has been confirmed by the ‘ACTwatch’ study, a 5-year multi-country research project launched in 2008 by a partnership between the London School of Hygiene and Tropical Medicine (LSHTM) and Population Services International (PSI) (O’Connell *et al*. 2011).

Due to the poor availability of ACTs among private retailers, children treated in the private sector were less likely to receive ACTs than children treated in the public sector. In Sierra Leone, children were likely to not have access to ACTs, because only 12% of children suspected of having malaria used public health services within 24 hr of the onset of symptoms (Amuasi *et al*. 2012). The ‘ACTwatch’ study reported that children treated in the private sector received non-artemisinin monotherapies at a rate ranging from 63 to 93%, varying according to country (Littrell *et al*. 2011). Even if this difference could be partially due to poor providers’ knowledge of malaria treatment, expensive ACTs and lack of efficient supply chains do not provide incentives for private retailers to stock ACTs (Arrow *et al*. 2004; Bosman and Mendis 2007).

#### Price

In the private sector, ACTs, when available, are likely to be more expensive than common monotherapies. Arrow estimated that without a global subsidy, the projected price of ACTs will be 5–10 times higher than the average price of chloroquine (Arrow *et al*. 2004). Chloroquine was first introduced in 1945, and its widespread use has increased its availability through well-consolidated supply chains. Its cheap price represents a barrier to the uptake of new ACTs by private retailers (WHO 2001). In Sierra Leone, in 2009, the price of ACTs was from 10 to 40 times higher than the prices for chloroquine or quinine according to Amuasi *et al.* (2012), or 5 to 24 times higher than prices of monotherapies according to the ‘ACTwatch’ study (Littrell *et al.* 2011; O’Connell *et al.* 2011). In Uganda, in 2007, ACTs were simply unaffordable for most of the population (Medicines for Malaria Venture 2008).

To summarize, as most of the affected populations worldwide relied on the private sector for antimalarial treatment, most people suffering from malaria simply did not have access to effective and cheap antimalarial treatment. Injecting ACTs into the public sector was not sufficient, because poor people suffering from malaria were most likely to look for treatments in private drug outlets, which are likely to stock cheap and ineffective monotherapies or expensive ACTs (Medicines for Malaria Venture 2008; O’Connell *et al.* 2011).

Penetration of the private sector by high-quality and affordable ACTs seemed difficult without an intervention. In January 2007, the Dutch Ministry of External Affairs held a meeting in Amsterdam with international stakeholders and decision makers. The idea of a global subsidy of ACTs was discussed again and renamed the ‘Affordable Medicine Facilities-malaria’ programme (Laxminarayan and Gelband 2009). In the face of strong opposition, two studies were conducted to pilot the introduction of subsidized ACTs in the private sector, the first in Tanzania in 2007–08, and the second in Uganda in 2008–09 (Medicines for Malaria Venture 2008; Samarasekera 2008; Sabot *et al.* 2009; Talisuna *et al.* 2009; Cohen *et al.* 2010; Rutta *et al*. 2011; Talisuna *et al.* 2012). The Clinton Health Access Initiative (CHAI), thanks to funds from the Bill and Melinda Gates Foundation, conducted the pilot study in Tanzania with encouraging results, even though it showed that the subsidy was not reaching the poorest of the poor (Samarasekera 2008; Laxminarayan and Gelband 2009; Sabot *et al.* 2009; CHAI 2009; Cohen *et al.* 2010; Rutta *et al*. 2011). Encouraging results also came from the Uganda study and from a mathematical model sponsored by The World Bank that compared the development of artemisinin resistance in presence/absence of subsidized ACTs and concluded that even a partial subsidy that was able to crowd out AMTs would be preferable to a delay in implementation (Laxminarayan *et al.* 2006; Laxminarayan and Gelband 2009).

The opponents to a global subsidy of ACTs argued that even with a subsidy, ACT prices would still be unaffordable for many poor people, and under-dosing could be a risk because people may start a course of treatment, only to stop prematurely because they could not afford the rest of the treatment (Frost and Reich 2009; Kamal-Yanni 2010; Kangwana *et al.* 2011). Ultimately, according to the opponents’ argument, this failure to complete treatment could lead to an increase in resistance to artemisinin (Laxminarayan *et al.* 2006; Laxminarayan and Gelband 2009). Furthermore, the opponents claimed that a subsidy that injects ACTs at the top of the supply chain could not effectively reach the most remote areas where poor people live and where malaria endemicity is higher (Arrow *et al.* 2004; Whitty *et al.* 2008; Cohen *et al.* 2010; Patouillard *et al.* 2010; Kedenge *et al.* 2013). But despite the firm stance of opponents, the AMFm managed to collect a large enough consensus to move forward, and in November 2008 the board of the Global Fund to Fight AIDS, Tuberculosis and Malaria (GFATM) approved a plan for the fund to roll out the first phase of the AMFm between late 2010 and early 2011 in eight countries: Cambodia, Ghana, Kenya, Madagascar, Niger, Nigeria, Tanzania (mainland and Zanzibar) and Uganda (Laxminarayan and Gelband 2009). The AMFm’s stated objectives were to increase ACTs’ affordability, availability, and use, and to increase the market share of ACTs relative to AMTs. The evaluation was conducted between 6.5 and 15.5 months after the arrival of the first AMFm co-paid drugs in the respective country, between October 2010 and January 2012 (Schäferhoff and Yamey 2010; Independent Evaluation Team 2012).

**Figure 2.**
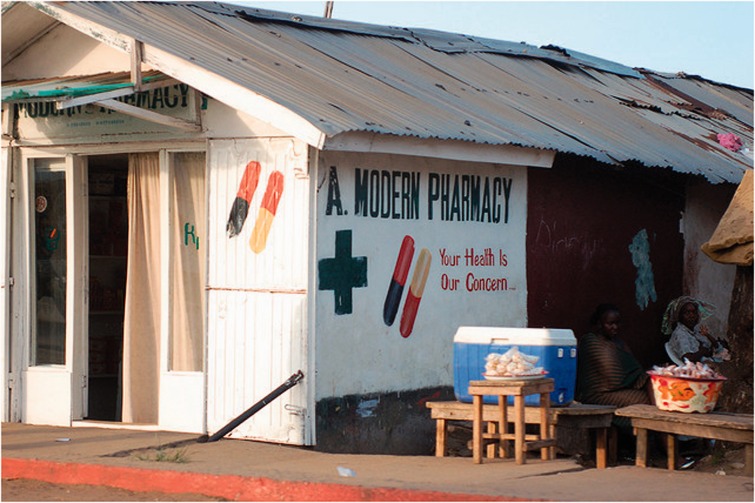
A private pharmacy in Monrovia, Liberia. Courtesy of The MENTOR Initiative, Liberia.

Apart from Uganda and Tanzania, which hosted the pilot studies, and apart from the eight countries enrolled in the AMFm, other Sub-Saharan countries, along with Myanmar, piloted the introduction of subsidized ACTs in the private sector: Angola, Cambodia, Cameroon, Democratic Republic of Congo, Liberia, Malawi, Rwanda, Senegal, Sierra Leone, and South Sudan (Figure 2; Schäferhoff and Yamey 2010). The results obtained in these countries, together with the independent evaluation of the AMFm Phase I, offer enough material for an overview of the global subsidy of ACTs after almost 10 years.

This review wishes to objectively depict the successes and challenges of this type of intervention, which has been at the center of the international debate concerning malaria control over the last decade. The public health community should value this opportunity to examine whether and how the subsidization of ACTs has actually delivered what was expected, i.e. better access to and affordability of ACTs. This review seeks to define what has worked and what has not within the global subsidy strategy; the resulting lessons should inform how we implement the next malaria control strategy currently under debate within the public health community: the introduction of rapid diagnostic tests (RDTs) into the private sector. The promise of RDTs lies in their ability to reduce over-treatment of non-malaria patients with ACTs by reducing the number of ACTs administered to non-malaria patients and to improve targeting of antimalarial treatment by increasing the number of ACTs administered to malaria-confirmed patients. Hence, RDTs represent a valuable tool for increasing accurate diagnoses of malaria in low-resource contexts, and the ultimate result of this increase in accurate diagnosing will be better malaria control (Yeung *et al.* 2011; Cohen *et al.* 2012a; Bastiaens *et al.* 2014).

## Methods

References for this non-systematic review were identified through searches of electronic databases, including PubMed, the Exerpta Medical Database (EMBASE), Cochrane Library Online, the WHO Library Information System (WHOLIS), the International Bibliography of the Social Science (IBSS), and Ovis. The following websites were also included: ACTwatch, ACT Consortium, Alliance for Health Policy and Systems Research, The Bill and Melinda Gates Foundation, CHAI, Foundation for Innovative New Diagnostics (FIND), Malaria Consortium, Management Sciences for Health, PSI, Roll Back Malaria, UNICEF, WHO and Google Scholar. The following search terms were used: ‘subsidized ACTs’, ‘subsidy malaria’ and ‘AMFm’. The timeline criterion for the search was from January 1971 to January 2014. Articles resulting from these searches and relevant references cited in those articles were reviewed. Articles published in English, French, Spanish and Portuguese were included.

## Results and discussion

### Main outcomes of the global subsidy of ACTs

Several outcomes can be used to evaluate the global subsidy of ACTs; the most relevant ones are availability and affordability of ACTs, and crowding out of monotherapies.

#### Availability of ACTs

According to the WHO, demand for ACTs reached an estimated 287 million treatments in 2011, a 32% increase from 2010. This surprisingly high demand was driven by ACTs procured by the private sector that recorded an almost 10-fold increase: the AMFm and other pilot projects were the main protagonists of this increase in demand (WHO 2011a).

Findings from pilot studies in Angola, Senegal, Kenya, Uganda, Tanzania and Nigeria indicate that availability of ACTs was remarkably increased after 1 year (Sabot *et al.* 2009; Uzochukwu *et al.* 2010; Kangwana *et al.* 2011; Talisuna *et al.* 2012; Kedenge *et al.* 2013). As pointed out by Yadav *et al.* (2012), the AMFm had to answer one key question: could the results of these smaller-scale projects be obtained at national levels? According to the evaluation of Phase I, the AMFm has succeeded in being a ‘game-changer’, having created an impressive increase in the availability of ACTs in the private sector in all of the countries where it was implemented, except Niger and Madagascar (Independent Evaluation Team 2012; Malm *et al.* 2013). However, it was generally found that even if availability of ACTs was higher at end-line than at baseline, it was still less than expected. For example, in Cambodia, the first country to introduce subsidized ACTs in the private sector in 2002, availability of ACTs rose from 40 to 63% after an implementation period of 5 years: not a very satisfactory result, according to the authors (Kheang *et al.* 2011; Yeung *et al.* 2011).

These findings might suggest that under trial conditions it is possible to rapidly increase availability of ACTs, but it is unclear whether the same results will be obtained at a national level. More importantly, since pilot studies are dependent on external funding, it is unclear if there exists a sustainable mechanism that can support a global subsidy of ACTs at a national level (Schäferhoff and Yamey 2010).

Overall the private sector subsidy programmes of ACTs seem to have been successful in increasing availability of effective antimalarial treatment in private outlets, but in some contexts this availability was limited if implementation was not supported by complementary interventions like community awareness campaigns (Yeung *et al.* 2011; Kedenge *et al.* 2013; O’Meara *et al.* 2013). It was also observed that other factors may have affected availability of ACTs in the private sector, such as the distance from the outlet to a public health facility carrying ACTs, the number of nearby private outlets stocking ACTs, and the volume of sales of the outlets themselves. All of these factors may contribute to stock more or less ACTs and ultimately affect their overall availability (Cohen *et al.* 2010).

#### Affordability of ACTs

In most of the pilot studies, ACTs were subsidized with a recommended price, and no fixed price was enforced. The evaluation of Phase I of the AMFm states that the benchmark of affordability (defined by a list of six indicators that include median costs to patients, median percentage markup between purchase and retail selling price, and median total markup from first-line buyer purchase price to retail selling price) was reached in six out of eight participating countries (Independent Evaluation Team 2012; Oxfam 2012). Large and significant decreases in the median price of ACTs were recorded, in these countries with a decline that ranged $1.28–$4.82 (Independent Evaluation Team 2012). In Ghana, prices of ACTs dropped 10-fold for treatment for both adults and children (Malm *et al.* 2013). In the first year of a national programme in Tanzania, they were reduced by 25%, but in Uganda there was no significant change in ACTs price (Sabot *et al.* 2009; Yadav *et al.* 2012; Fink *et al.* 2013). ACTs tended to be slightly more expensive in urban than rural areas, with some exceptions (Schäferhoff and Yamey 2010).

Despite these encouraging results, according to data from ACTwatch, in Madagascar, Nigeria and Uganda, median prices of ACTs were still 5–24 times higher than prices of monotherapies (O’Connell *et al.* 2011). In Tanzania, prices of ACTs were still higher than median prices of amodiaquine, and in Uganda, ACTs still cost twice as much as quinine (Sabot *et al.* 2009; Yadav *et al.* 2012; Fink *et al.* 2013). These results are in contrast with what has been reported by Smith *et al.*, who affirms that in Kenya, 5 months after the beginning of the AMFm, non-subsidized and subsidized ACTs were one-half to two-thirds less expensive, respectively, than antimalarial monotherapies. The reasons for those differences are probably due to different mechanisms and actors in the private-market supply chain (Smith *et al.* 2011).

Overall the private sector subsidy programmes of ACTs were not effective in reducing prices of ACTs other than for the ones it co-paid; affordability of ACTs was indeed increased, thanks to the availability of subsidized ACTs, but a subsidy is still needed to keep prices low (Davis *et al.* 2013).

In some scenarios, a price that is too affordable can be seen as a constraint. For decades chloroquine was an effective and cheap drug, but poor people could not afford to buy a full course every time they needed it, with the result that resistance developed and ultimately rendered a good drug useless. But despite concerns that resistance to ACTs may develop for the same reasons, it is still unlikely that in the absence of the AMFm the market will be invaded by excessively affordable ACTs; prices of non-subsidized ACTs are likely to remain higher than the average price of AMTs and chloroquine or other ineffective monotherapies (Kamal-Yanni 2010).

#### Crowding out antimalarial monotherapies

Uganda was one of the countries that saw a significant effect of the subsidy of ACTs in crowding out monotherapies. From September 2008 to May 2010 the market share of ACTs in the country increased from 1 to 69%, while quinine and chloroquine market share decreased from 90 to 29% (Talisuna *et al.* 2009; Talisuna *et al.* 2012). In the two countries where availability of AMTs was >5% at baseline (Nigeria and Zanzibar), the AMFm was effective in reducing the market share of oral AMTs (Tougher *et al.* 2012). Nonetheless, use of monotherapies was still high after years of subsidized ACTs in several countries. The subsidy of ACTs was effective in increasing availability of ACTs in the private sector, but it showed little effect in reducing the market share of ineffective monotherapies and AMTs (Sabot *et al.* 2009; Littrell *et al.* 2011; Smith *et al.* 2011; Amuasi *et al.* 2012; Independent Evaluation Team 2012; Oxfam 2012; Talisuna *et al.* 2012; Tougher *et al.* 2012; Cohen *et al.* 2013).

The reason for this can be found in the levels of providers’ knowledge about malaria treatment. Increasing providers’ knowledge of malaria treatment is essential, along with subsidization of ACTs. When providers are trained in malaria case management, use of ACTs increases, and use of monotherapies decreases. Trained providers are also more likely to stock ACTs and promote them to clients (Cohen *et al.* 2010; O’Connell *et al.* 2011; Kheang *et al.* 2011; Yeung *et al.* 2011; Talisuna *et al.* 2012; Kedenge *et al.* 2013; O’Meara *et al.* 2013). BCC activities are fundamental to increasing uptake of ACTs, and they have to be targeted to both providers and costumers; campaigns that reach customers influence providers as well (O’Connell *et al.* 2011; Klein *et al.* 2012; Oxfam 2012; Talisuna *et al.* 2012). BCC campaigns were effective in Cambodia, increasing customers’ awareness of ACTs from 47 to 98% in 5 years; in Uganda and in Kenya, they have resulted in substantial increases in health-seeking behaviour in children under 5 years of age within the first 24 hr of symptoms (Yeung *et al.* 2011; Talisuna *et al*. 2012).

### Challenges from the field

The private sector subsidy programmes of ACTs have always attracted a modest number of opponents whose main concern is the cost-effectiveness of the strategy and who warn about its role in accelerating the emergence of resistance to artemisinin (Bosman and Mendis 2007; Whitty *et al.* 2008; Laxminarayan and Gelband 2009; Kamal-Yanni 2010). After 10 years of interventions, the results indicate that these concerns were not unreasonable, as affordable and available ACTs have been shown to pose some serious risks to public health (Kheang *et al.* 2011; Yeung *et al.* 2011).

#### Over-treatment

Central among the concerns of opponents to ACTs is the fear that a price subsidy might encourage over-treatment with ACTs. Subsidization of ACTs represents an incentive for consumers to use cheap ACTs preventively, without a confirmed diagnosis of malaria; and because most fevers are non-malarial, there is a chance that unnecessary sales of ACTs will increase, resulting in poor treatment targeting (Whitty *et al.* 2008; Kangwana *et al.* 2011; Ajayi *et al.* 2013). Data from the 2011 World Malaria Report reported that the number of ACTs administered is much higher than the number of confirmed cases of malaria in most of the countries in which ACTs are subsidized in the private sector (WHO 2011 b). As most of the malaria countries targeted by private sector subsidy programmes of ACTs are not yet in a pre-elimination phase in malaria control, some may argue that in these over-treatment scenarios offer interesting up-sides to control malaria mortality, especially in the highly vulnerable groups. However, data from the WHO shows that there has been a reduction in malaria incidence worldwide, in which case over-treatment with ACTs could sustain poor targeting (WHO 2011 b). Even more concerning is that other diseases whose burden is increasing in Sub-Saharan Africa, particularly respiratory infectious diseases, have many symptoms similar to those of uncomplicated malaria, and as a result many patients reporting fever are not being treated for the correct disease (Oxfam 2012; UNICEF 2012). The subsidy of ACTs may lead to a scenario where ACTs are too readily available and affordable, which in turn may increase the risk that patients over-consume ACTs without investigating the real causes of the symptoms that led them to a private retailer in the first place.

The effect of over-treatment on accelerating the emergence of resistance to artemisinin seems to be controversial. The mathematical model used by The World Bank in 2006 shows convincingly that a subsidy of ACTs is likely to slow the development of resistance to artemisinin-based treatments, even if such a subsidy were to increase ACT use significantly (Laxminarayan *et al.* 2006). This model demonstrates that over-consumption of ACTs is unlikely to increase resistance to artemisinin (Whitty *et al.* 2008; Kangwana *et al.* 2011). Unfortunately, the model did not take into account under-dosing.

#### Under-dosing

Under-dosing of ACTs is an express ticket towards resistance to artemisinin. Many ACTs require different dosages for children and adults but have the same formulation; to keep manufacturers’ prices low, formulations that have the same strength (i.e. artemether 20 mg and lumefantrine 120 mg) are combined during the packaging process to obtain different dosages. For example, blisters of Coartem for adults manufactured by Novartis have 24 pills, four times the number of pills in Coartem blisters for children weighing 5–15 kg. Such packages of subsidized ACTs encourage easily division of the drugs among patients, which leads to under-dosing (Oxfam 2012).

The mathematical model conducted by The World Bank did not take into account under-dosing, nor did it evaluate how much this factor contributes to the development of resistance to artemisinin (Laxminarayan *et al.* 2006). This is unfortunate, because we know that under-dosing is common in poor populations—and with increased sales of ACTs, more of those who use the treatment are likely to not complete the full course (Schäferhoff and Yamey 2010). Low prices could boost unnecessary sales of ACTs and thus unnecessary uptake of artemisinin at lower doses. Furthermore, there is a risk that the ACTs available in the market are not quality-assured and that the artemisinin contained in the formulation is at a sub-therapeutic dose, contributing to the threat of artemisinin resistance (Whitty *et al.* 2008; Kamal-Yanni 2010; Cohen *et al.* 2012 b). Despite intensive BCC campaigns in Cambodia, for example, under-dosing was highly reported by customers and providers even after years of subsidy programmes (Kheang *et al.* 2011; Yeung *et al.* 2011). The issue of under-dosing is central, and in the long term it could undermine one of the goals of private sector subsidy programmes of ACTs by accelerating the development of resistance to artemisinin.

#### Coverage

The difficulties of treatment reaching remote and less populated areas became a major issue as soon as Arrow’s work was published thanks to an analysis of the mechanisms regulating supply chains conducted by Patouillard *et al.* (2010). The capacity of the private sector subsidy programmes of ACTs to correctly target the people who need them most were a subject of intense debate. Some argued that the subsidy would be captured by middlemen in the distribution chain and that market power in the private sector would have reduced affordability of and access to ACTs (Arrow *et al.* 2004; Whitty *et al.* 2008; Cohen *et al.* 2010; Patouillard *et al.* 2010; O’Connell *et al.* 2011; Kedenge *et al.* 2013). The low efficiency of the subsidy in reaching remote areas was confirmed by the pilot study in Tanzania, studies in Uganda and the ‘ACTwatch’ study (Sabot *et al.* 2009; Littrell *et al.* 2011; O’Connell *et al.* 2011; Yadav *et al.* 2012). There were considerable and persistent geographical disparities in the availability of ACTs between high- and low-population areas. In Sierra Leone, people most in need of effective and cheap antimalarial treatments were located in hard-to-reach areas, where 83% of retailers did not have a large enough incentive to stock ACTs, and where non-policy antimalarials were offered at a much cheaper price (Amuasi *et al.* 2012).

At the beginning of the AMFm, studies in Kenya and Tanzania investigated factors that influence the likelihood of stocking ACTs in the private sector (O’Meara *et al.* 2013). Several factors were identified, including competition, customer demand and higher volume of sales of ACTs at nearby outlets, as driving forces behind the stocking of ACTs (Larson *et al.* 2013). Other factors might contribute to decreases in the likelihood of stocking ACTs, such as proximity to public HFs and location in remote areas (O’Meara *et al.* 2013). Supporters of private sector subsidy programmes argued that other complementary interventions, such as community awareness campaigns and/or incentives to retailers to stock ACTs, are needed to promote stocking ACTs in remote areas (Cohen *et al.* 2013; O’Meara *et al.* 2013). However, none of these methods for increasing access to ACTs in rural areas seems to have been routinely implemented.

#### The importance of complementary activities

**Figure 3.**
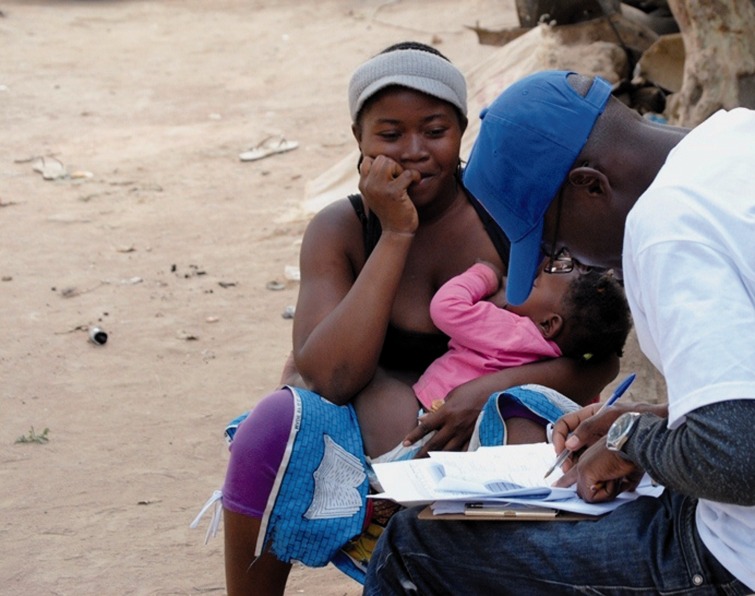
In-depth interviews with customers in Huambo province, Angola. Courtesy of PSI, Angola.

Complementary activities have succeeded in increasing providers’ and customers’ awareness of ACTs and have been widely advocated by several stakeholders (Figure 3; Yeung *et al.* 2011; Ajayi *et al.* 2013; Talisuna *et al.* 2012; Kedenge *et al.* 2013; O’Meara *et al.* 2013; Willey *et al.* 2014). From the literature on the subsidy of ACTs, it is clear that the need for interventions that address a full range of topics beyond a price subsidy of ACTs, such as improving providers’ and customers’ knowledge of malaria treatment, publicizing the risks of under-dosing, and incentivizing supplies in remote areas, is of great importance (O’Connell *et al.* 2011). Interventions should also strengthen demand creation through BCC activities to encourage uptake of ACTs (Ajayi *et al.* 2013).

#### Sustainability

The subsidy aimed to stimulate private companies’ production of ACTs and to crowd in the market with a number of effective antimalarial treatments, which in turn would create competition and reduce the average price of ACTs. This decline in price would have made the mechanism sustainable in the long term and independent of external funding (Bate and Hesse 2009). Several years later, results showed that the private sector subsidy programmes of ACTs had a modest effect in reducing prices of ACTs and increasing affordability of non-subsidized ACTs (Independent Evaluation Team 2012).

It is unclear what change is taking place that requires the replacement of international donors and NGOs. Is subsidy ending? Will wholesalers and retailers buy ACTs through their own channels and remove monotherapies from their original supply chains? Will the income be high enough to incentivize sales of ACTs and to reduce availability of monotherapies? To date, it is not clear who will assume the responsibility for providing effective and affordable antimalarial treatments (Oxfam 2012).

The private sector subsidy programmes of ACTs were effective overall in achieving the ambitious benchmark of increasing availability of ACTs in the private sector in a considerable number of countries, but few studies have demonstrated its effectiveness in crowding out monotherapies or increasing affordability of ACTs (Schäferhoff and Yamey 2010; Bump and Fan 2012; Independent Evaluation Team 2012; Talisuna *et al.* 2012; Tougher *et al.* 2012; O’Meara *et al.* 2013; von Schoen-Angerer 2013). Some may say that the up-side of increasing ACTs’ availability in the private sector may not be enough to compensate the down-side of the failure to crowd out monotherapies (Kamal-Yanni 2010). Even more concerning is that affordable and available ACTs are sold in the absence of a malaria diagnosis. This leads to unnecessary sales of ACTs, which will lead in turn to a rise in the unnecessary uptake of artemisinin at lower doses and, ultimately, greater resistance to artemisinin (De Allegri *et al.* 2011).

However, as Arrow states, the subsidy of ACTs is not the answer to controlling malaria cases. The aim of the subsidy was to preserve the effectiveness of antimalarials for as many citizens as possible and to buy time for the development of new effective compounds (Arrow *et al.* 2004; Sabot *et al.* 2009). Considering the subsidy’s success in buying almost 10 years of time, Arrow’s goals may have been met; the international community, on the other hand, should have been more efficient in promoting all of the complementary interventions that he proposed as part of the subsidy strategy in addition to treatments.

### Towards a subsidy of RDTs: building on lessons learned

The public health community now agrees that the private sector subsidy programmes of ACTs have caused excessive orders of ACTs, the consumption of which was not based on clinical need (irrational use). Increased sales of ACTs do not necessarily mean increased malaria treatment, and we cannot know how many malaria cases were effectively treated thanks to the private sector subsidy programmes of ACTs (WHO 2011a; Oxfam 2012). As already noted, incorrect use of ACTs may result in the development of resistance to artemisinin. Cambodia stands out as the first country to introduce subsidized ACTs in the private sector and also the first country to record resistance to artemisinin in 2008 (Noedl *et al.* 2008). Although these two events may not be related, there is still a high chance that the combination of over-treatment and under-dosing will increase the likelihood of resistance development.

In 2011, the WHO released a new policy on malaria case management, emphasizing the need for a rapid shift from malaria treatment to malaria diagnosis. The policy recommended parasitological confirmation by microscopy or RDTs for all patients with suspected malaria before the start of treatment (WHO 2011a). The impetus for this policy is the worryingly high number of unnecessary sales of ACTs; it aims to reduce the gap between malaria-treated cases and malaria-confirmed cases in order to avoid administration of antimalarials when the causes of fever are other than malaria (Uzochukwu *et al.* 2010).

Recently, countries have been encouraged, as part of the interventions to be implemented alongside the introduction of subsidized ACTs into private markets, to increase use and affordability of diagnostics (The Global Subsidies Initiative 2010). RDTs have been widely advocated by governments and the international community as cost-effective tools for malaria diagnosis, and they are now regarded as essential for ensuring that ACTs are given to malaria-confirmed patients (Uzochukwu *et al.* 2010). RDTs have been shown to increase accurate malaria diagnoses in contexts of low human and financial resources, which will ultimately result in better treatment targeting (Cohen *et al.* 2012a; Bastiaens *et al.* 2014).

To increase malaria diagnosis using RDTs, several strategies have been proposed, including subsidization of RDTs in the private sector a method similar to the subsidy of ACTs (Yeung *et al.* 2011; WHO 2011a; GFTAM 2010). A randomized controlled trial developed by Cohen justifies the use of RDTs in the private sector to reduce both under- and over-treatment, at least in the long term when subsidized ACTs are available on the same counter (Cohen *et al.* 2014). However, use of RDTs may bolster the arguments of sceptics of ACT subsidization, because private retailers may not have an incentive to sell cheap diagnostic tests that will result in fewer drugs purchased. Other key questions surrounding a subsidy of RDTs in the private sector: if malaria test results are negative, will private retailers have the ability to test for other causes of malaria-like symptoms? If not, will the system rely on referrals to public HFs? Will private retailers prefer to sell other treatments, like antibiotics? And above all, how can RDTs be made affordable to the public and still rentable to private retailers, without costing more than ACTs or ineffective antimalarial treatments (Cohen *et al.* 2014)?

Despite these many questions, several countries have already hosted pilot studies to introduce RDTs in the private sector, and it is likely that others will follow (Albertini *et al.* 2012; Cohen *et al.* 2012a; Kizito *et al.* 2012; Malaria Care 2013; Bastiaens *et al.* 2014; UNITAID 2014). The private sector subsidy programmes of ACTs has offered us enough material to test the effectiveness of a subsidy of health commodities, and we have to be as wise as possible in applying the lessons we learned from the subsidy of ACTs to a potential subsidy of RDTs.

#### Availability

RDTs could make a crucial difference especially in rural areas that lack human and economic resources. The private sector subsidy programmes of ACTs have started to open the road to subsidization of commodities through existing supply channels with the goal of reaching a target population (Patouillard *et al.* 2010; Kedenge *et al.* 2013). A subsidy of RDTs could follow the intervention channels established by subsidized ACTs, and local wholesalers and private retailers would make RDTs available in areas where ACTs are and where they are most needed. However, we have seen that subsidized ACTs were not 100% effective in reaching remote areas: this outcome suggests that the introduction of subsidized RDTs should also look beyond the channels and delivery mechanisms that have already been explored by the subsidy of ACTs (Sabot *et al.* 2009; Littrell *et al.* 2011; O’Connell *et al.* 2011; Yadav *et al.* 2012; Fink *et al.* 2013).

On the other hand, if the private-sector channels used to introduce subsidized RDTs are different from those used to introduce ACTs, there is the risk that subsidized RDTs and ACTs may not be available at the same time and in the same place. This may have undesirable consequences because:

- RDTs sold in the absence of affordable ACTs may result in increased uptake of AMTs or ineffective antimalarial treatments in cases of positive test results for malaria,
- availability of ACTs in the absence of RDTs may result in over-treatment and poor targeting, while at the same time it may divert clients to use RDTs available at nearby facilities or outlets.

In the past, the high malaria mortality rate in vulnerable sub-groups of the population (like children under five) has been a great incentive to introduce subsidized ACTs (Amuasi *et al.* 2012). Because the susceptibility of this group remains high, the subsidy of RDTs should not serve as a substitute for available, effective and affordable ACTs in all endemic areas. The two interventions should be conceived and implemented simultaneously, especially in countries where the ACT market is not yet satisfactory.

#### Affordability

The subsidy of ACTs has been shown to be effective in reducing the price paid by end-consumers for a subsidized treatment course of ACTs (Schäferhoff and Yamey 2010; Independent Evaluation Team 2012). In order to incentivize correct use of malarial treatment and improve treatment targeting, RDTs should be cheaper than ACTs: this is likely to happen only if RDTs are subsidized. The price scheme of subsidization of RDTs is central: to increase their uptake by the population, RDTs should cost less than any other antimalarial treatment; at the same time, they should offer some income to private retailers and physicians in order to incentivize their use. Thus, there may be an interest in linking the subsidy of RDTs to the price scheme of ACTs, as these two interventions are all but independent. If these two interventions are implemented separately, there is the risk that one could undermine the other. RDTs that are more expensive than ACTs are counterproductive, because people might be incentivized to buy malaria treatment without prior diagnosis. On the other hand, RDTs that are far less expensive than ACTs might not offer enough income to private retailers and physicians to incentivize their use. Furthermore, RDTs are time-consuming, and since many of them will produce negative test results, they will be viewed as responsible for fewer sales of the more rentable ACTs. Thus, when introducing subsidized RDTs in the private sector, a market assessment of availability and pricing of ACTs is desirable in order to determine the best pricing scheme for RDTs. At the same time, we should avoid increasing the price of ACTs, because people with medical prescriptions for malaria still need access to affordable and effective antimalarial treatment.

#### The regulatory environment

**Figure 4.**
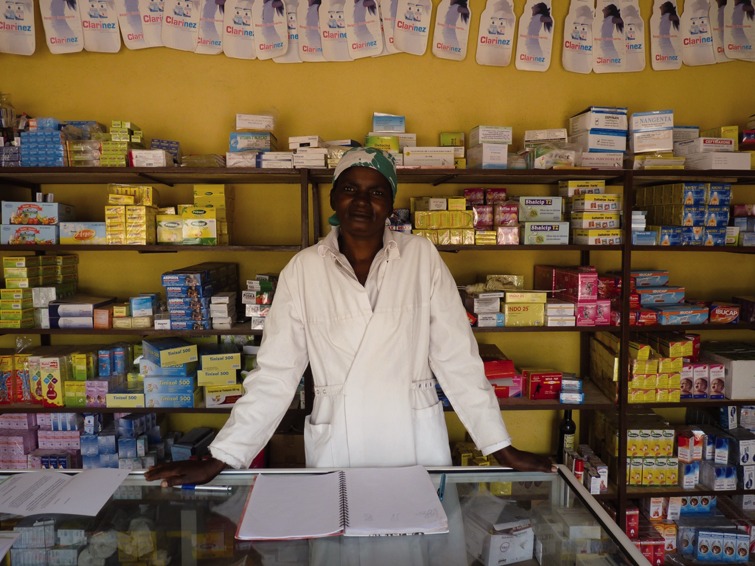
Worker from a private pharmacy in Kampala, Uganda. Courtesy of PSI, Uganda.

Many concerns have been raised in relation to the need to capacitate private health workers for safe performance of RDTs (Albertini *et al.* 2012; Cohen *et al.* 2012a). The private sector is populated with sellers (of both drugs and of other products and services), pharmacists and private physicians who in some cases do not have the required skills to perform RDTs safely (Figure 4). In contrast to ACTs, RDTs are not a product to be sold, but rather a service to be promoted. In addition to the challenges to RDT distribution already mentioned, many private sellers may be reluctant to carry RDTs because of the additional medical action required of them. Cases with negative test results for malaria should be referred to public HFs or private physicians for further investigation of the causes of fever and other malaria-like symptoms. Finally, nurses and physicians may be resistant to pharmacists and drug sellers performing RDTs at their outlets, which resistance may be a barrier to the uptake of RDTs in the private sector.

All of these issues must be addressed at the level of regulatory institutions prior the introduction of RDTs in the private market. The subsidy of ACTs presented an opportunity to strengthen partnerships between international organizations and local institutions for training of private providers like physicians, pharmacists and drug sellers in malaria treatment (Willey *et al.* 2014). Pilot projects and programmes for RDTs may likewise count on already existing partnerships between organizations of private health providers and the government for training in malaria diagnosis.

Regulatory bodies should create a fertile ground for RDTs in the private sector by:

- identifying which types and brands of RDTs are allowed in which countries;
- determining the quality assurance and quality control standards to which RDTs in the private sector must adhere;
- specifying the minimum requirements for the purchase, storage, performance and sale of RDTs by private outlets/providers (storage conditions, minimum space requirements, etc.);
- outlining a training curriculum on malaria diagnosis to be held by the local NMCP and MoH as well as minimum requirements for private providers to be entitled to perform RDTs at their outlets and facilities;
- clearly defining the roles and responsibilities of the different actors in the private sector who will perform RDTs;
- outlining the roles and responsibilities of public and private HFs in the referral system and in the biological waste management system that must be put in place for the introduction of RDTs in the private sector.

Timing of policies and strong advocacy can do much to promote correct use and ultimately cost-effectiveness of RDTs in the private sector.

#### The importance of complementary activities

When allocating funds to introduce RDTs in the private sector, a considerable share should go towards reinforcement of all complementary activities that increase correct uptake of RDTs by the population and safe use by private providers. The private sector subsidy programmes of ACTs have demonstrated that these interventions are of paramount importance (Yeung *et al.* 2011; Ajayi *et al.* 2013; Talisuna *et al.* 2012; Kedenge *et al.* 2013; O’Meara *et al.* 2013; Willey *et al.* 2014). Whereas subsidization of health commodities represents the core of the intervention itself, complementary activities are the basis for the intervention’s success. When a commodity is introduced in the private sector, it is not only the single commodity that is introduced (the ‘what’), but all of the behaviours and knowledge that surround it (the ‘how’) as well. Possibly the most rewarding lesson gained from the private sector subsidy programmes of ACTs is that the ‘what’ should always be introduced alongside the ‘how’. After the selection of the health commodity to be introduced and the determination of its introductory price, other activities such as provider BCC campaigns, on-the-job training and supervision should drive how, when, and where providers use it. Providers should also be regularly monitored and evaluated with respect to their use of the subsidized commodity in order to continuously promote correct behaviour.

Complementary activities involving providers should be in parallel with a fertile active regulation: there is indeed an opportunity to promote a correct behaviour (test before treat) among providers by banning an incorrect one (treatment without prior positive diagnosis of malaria). We have seen that poor targeting is a persistent problem and that there is a need to urgently reverse the under-dosing and over-treatment that the private sector subsidy programmes of ACTs may have unintentionally stimulated. Complementary activities involving providers could offer a valuable means to improve correct targeting.

Some may argue that better targeting of antimalarials may come at the price of misuse of antibiotics, because providers may be likely to prescribe antibiotics in cases of negative RDT results. For this, complementary activities involving providers again represent an excellent means to promote providers’ capacity building and improve their skills and knowledge in fever case management.

Complementary activities should be directed to both customers and providers. Awareness and BCC campaigns may be helpful in increasing a population’s uptake of and demand for RDTs by reinforcing the importance of testing before treating. If BCC campaigns with the population are under-evaluated when planning a programme that introduces RDTs in the private sector, there is the risk that providers who are aware of the importance of testing before treating could find a lack of trust in the population towards RDTs. Providers should not be left alone in promoting malaria diagnosis among their clients. For that reason, BCC and marketing campaigns have to be implemented alongside the subsidy of RDTs.

We encourage donors and funding agencies to allocate a considerable amount of funds to these complementary activities, which must be mandatory in projects that subsidize RDTs and ACTs in the private sector. A subsidy of RDTs could become one of the most effective interventions to efficiently target malaria treatment and further delay development of resistance to artemisinin, if only both access and demand are considered fundamental components of the same strategy.

#### Sustainability

Considering the current uncertainty regarding the future of the AMFm programme to provide affordable ACTs to the private sectors of malaria-endemic countries, there is the risk that subsidized RDTs will not lead to an RDT market that is appealing to manufacturers, either (Oxfam 2012). In the absence of such stimulation, doubts have been raised around the sustainability of such an intervention beyond the projects or programmes that promote it. Unfortunately, the only way to sustainability—making RDTs economically feasible in the private sector—seems to be the hardest one. If we have a fertile policy environment accompanied by BCC campaigns that promote the uptake of RDTs by private providers and demand from customers, there may be a chance that the private sector will become fully engaged in manufacturing high-quality RDTs. This will expand the market of RDTs in malaria-endemic countries to the point that it will be large enough to stimulate competition.

The introduction of RDTs in the private sector requires an interactive and multi-layered system that involves several actors, such as organizations of private professionals that assure capillary communication to their affiliates, public institutions that provide continuous training of private providers to cope with high staff turnover and supervise adherence to national guidelines, local entities that support disposal of biological waste, international NGOs and/or a civil society to promote BCC campaigns, and regulatory bodies that ensure that a consolidated RDT quality assurance and quality control system is in place in the respective country. Because it is unlikely that all of these interventions will be handed over entirely to either the private or the public sector in the short term, we should concentrate our efforts on making the RDT price scheme sustainable in the short term. In this way, we will aim to create the conditions for sustained demand of RDTs from both providers and customers by advocating policies that emphasize diagnosis of malaria before treatment and by implementing BCC campaigns that increase demand for RDTs from customers. This change in approach will ultimately deliver a well-consolidated system where RDTs are part of day-to-day activities in healthcare for both providers and customers; this system will increase competition, reduce prices of RDTs in the long term and leverage the private and public sector to take full responsibility for a real need—testing for malaria before treating it.

## Conclusion

Although it may sound logical, in the context of malaria control, to increase availability of diagnostics before increasing availability of treatments, this was not possible at the time of the private sector subsidy programmes of ACTs because RDTs were not yet available on a large scale. This was the reason why subsidized ACTs were introduced first, along with the fact that increasing access to effective antimalarials was the fastest way to reduce malaria mortality. Little by little it became clear that increasing availability of malaria diagnostic methods would have been more effective for targeting of antimalarial treatments.

Now that more countries are starting to introduce RDTs in their private sectors, we should remember the lessons we learned from the private sector subsidy programmes of ACTs and effectively use the funds allocated to this intervention. The lessons learned here should be valued by policymakers in their planning of future interventions aimed to increase availability and affordability of RDTs in the private sector.

As is often the case with public health interventions, the results are not black and white; rather, our reading of them should take into account all of the complexity surrounding the implementation of such ambitious interventions. The successes and challenges of the subsidy of ACTs should be seen from several perspectives, from the international funders, to the suppliers of *A. annua*, to the local wholesalers, to private providers, to public health care workers and down to the final customer. Instead of viewing the subsidization of ACTs as a categorical success or failure, the public health community should acknowledge that the private sector subsidy programmes of ACTs have been an opportunity to learn what works and what does not when subsidized commodities are introduced into the private market.

We should now be brave enough to recognize this by introducing RDTs in the private sector. Their introduction will allow us to make the most out of the knowledge that we have gained and recognize that complementary activities, such as BCC campaigns, marketing campaigns, training and supervision are anything but unnecessary interventions in the introduction of RDTs in the private sector. Rather, they represent the sole fertile ground available to us for promotion of effective and correct malaria diagnosis in the private sector that will lead to correct fever case management.
